# Ratiometric near-infrared biosensors based on self-assembled nanoparticles and target-triggered signal amplification for sensitive detection of miRNAs

**DOI:** 10.1039/d5ra04934b

**Published:** 2025-11-27

**Authors:** Tingman Xu, Chengke Huang, Tiantian Ge, Songsong Song, Yanyi Tan

**Affiliations:** a Midu Medical Technology (Zhongshan) Co Ltd 528437 China xmiduyl@163.com; b College of Materials Science and Engineering, Fuzhou University Fuzhou 350108 China; c Department of Cardiology, North Campus of Zhongda Hospital Affiliated to Southeast University Nanjing 210008 China gttcardiology@163.com dr_lls@fjmu.edu.cn

## Abstract

The abnormal expression of microRNA-21 (miRNA-21) is highly correlated with acute myocardial infarction and can serve as a biomarker for its early diagnosis. In this study, a ratiometric fluorescent probe based on target-triggered signal amplification using upconversion nanoparticles (UCNPs) as energy donors and gold nanoparticles (AuNPs) as energy acceptors was fabricated for the detection of miRNA-21. First, hairpin DNA (H1) with sulfhydryl and amino groups at both terminations is connected to AuNPs and UCNPs through Au–S bonds and amide bonds, leading to the quenching of upconversion luminescence (UCL). The target miRNA-21 can trigger H1 and obtain a miRNA/H1 duplex structure, leading to AuNPs being far from the UCNPs and recovering the UCL. The opened H1 hairpin exposes fragments that can recognize another hairpin DNA (H2) labelled with Cy5, resulting in the target miRNA-21 being replaced by H2 and triggering catalytic hairpin assembly (CHA) signal magnification. The stationary fluorescence of Cy5 and the changing UCL of the UCNPs construct a ratiometric biosensor, which was used to detect miRNA-21, with a low detection limit (LOD) of 0.28 pM. Additionally, this biosensor can determine miRNA-21 in serum samples, with recoveries ranging from 94.3–102.7%, suggesting that the proposed method shows broad prospects in the sensitive and accurate detection of clinical sample biomarkers.

## Abstract 2

The abnormal expression of microRNA-21 (miRNA-21) is highly correlated with acute myocardial infarction and can serve as a biomarker for its early diagnosis.

## Graphical abstract

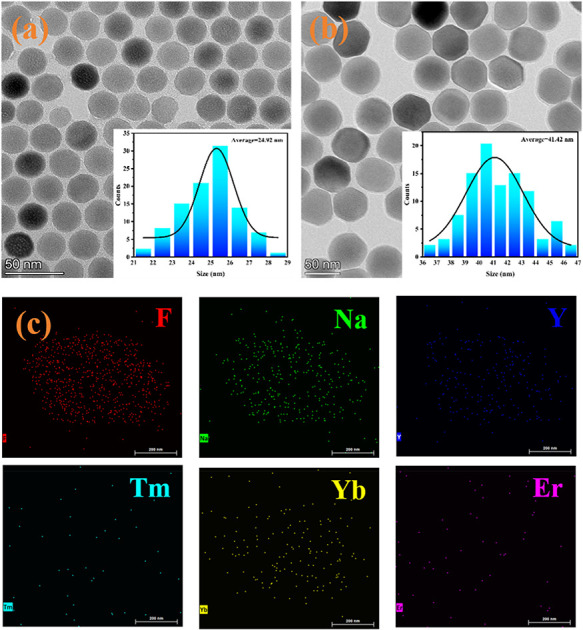

## 1 Introduction

MicroRNAs (miRNAs) are a family of short, single-stranded noncoding RNAs (18–24 nucleotides) that play a critical role in post-transcriptional gene regulation by modulating mRNA stability and translation.^1,2^ Although these regulatory molecules participate in essential cellular activities, their irregular production has been connected to multiple disorders.^3,4^ Specifically, abnormal expression of miRNA-21 is closely associated with acute myocardial infarction.^5^ Therefore, miRNA-21 has potential as a diagnostic indicator for acute myocardial infarction.^6,7^ However, the accurate quantification of miRNA-21 remains technically challenging due to its low physiological concentrations and sequence similarities with other miRNAs. To address this limitation, the development of highly sensitive and selective detection methods is essential for enabling early and reliable diagnosis of acute myocardial infarction.

Currently, reverse transcription-quantitative polymerase chain reaction (RT-qPCR),^8^ northern blotting,^9^ and microarrays^10^ are widely used for assessing miRNA expression levels.^11–13^ However, these approaches have several shortcomings, such as insufficient detection sensitivity, long analysis time, environmental interference susceptibility, and specialized apparatus requirements.^14,15^ Therefore, an innovative approach with high amplification efficiency, minimal interference, and excellent selectivity is urgently needed. A nucleic acid-mediated signal amplification is a notable means to increase determination sensitivity. The signal enhancement pathways primarily consist of catalytic hairpin assembly (CHA),^16–18^ rolling circle amplification (RCA),^19^ polymerase chain reaction (PCR),^20^ and hybridization chain reaction (HCR).^21^ Among them, CHA is a nonenzymatic signal amplification strategy that is mediated through DNA hybridization and toehold-mediated exchange. Therefore, CHA has several unique advantages, such as cost-effectiveness, high selectivity, and reduced background, and is widely applied for highly sensitive detection of the target biomolecules.^22–24^ These autocatalytic methods can enhance the signal and shorten the reaction time to some extent. However, for analytes with low expression in biological matrices, successive enhancement is still needed.

Several emerging approaches utilizing multiple functional nanomaterials and distinct mechanisms, such as fluorescence,^25,26^ surface-enhanced Raman spectroscopy,^27,28^ colorimetry,^29,30^ and electrochemistry,^31,32^ have been developed to detect miRNAs. Among them, fluorescence-based approaches are widely adopted for the quantification of miRNAs because of their exceptional sensitivity, straightforward protocol, and good repeatability.^33^ Numerous nanomaterials with luminescent properties, such as organic dyes,^34^ quantum dots (QDs),^35^ and upconversion nanoparticles (UCNPs),^36,37^ have been investigated for the quantification of miRNAs. Notably, UCNPs are anti-Stokes luminescent nanomaterials based on rare earth elements that can absorb near-infrared (NIR) radiation and generate intense visible light *via* multiple photoabsorption processes.^38^ These methods have several advantages, such as a high signal-to-noise ratio, high sensitivity, narrow bandwidth, and low phototoxicity.^39^ In particular, UCNPs, as energy donors, have been applied to develop fluorescence resonance energy transfer (FRET)-based biosensors.^40^ On the other hand, gold nanoparticles (AuNPs) can effectively quench fluorescence through an energy transfer phenomenon and thus are used as ideal energy acceptors for FRET-based biosensors owing to their adjustable optical characteristics, facile surface functionalization, good biological compatibility, and superior molar absorptivity.^41–43^ However, the designed biosensors based on UCNPs and AuNPs usually rely on absolute changes in fluorescence intensity and are vulnerable to variability from experimental conditions, such as excitation source fluctuations and temperature-dependent signal drift during application when applied to biological samples. In contrast, ratiometric fluorescence biosensors can eliminate the influence of environmental conditions by calculating the ratiometric fluorescence intensities at two different wavelengths. Therefore, constructing ratiometric fluorescence biosensors is a robust strategy to enhance detection reliability and avoid false positive signals.

**Scheme 1:**
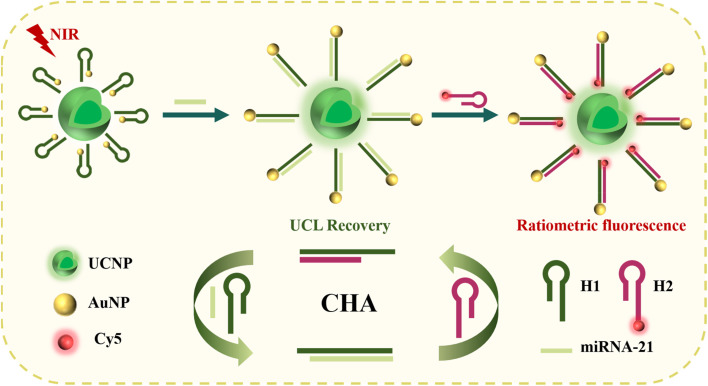
Schematic diagram of a fluorescence sensor for measuring miRNA-21.

Inspired by these strategies, we constructed a novel FRET-based sensitive biosensor for the quantification of miRNA-21, which combines CHA signal amplification and nanomaterials with AuNPs and UCNPs. As shown in Scheme 1, hairpin DNA (H1) with sulfhydryl and amino groups at both ends is conjugated to the AuNPs and the UCNPs through Au–S bonds and amide bonds. Consequently, the upconversion luminescence (UCL) of the UCNPs was effectively suppressed *via* FRET between the UCNPs and the AuNPs. When the target miRNA-21 is present, it can recognize H1 and trigger a strand replacement reaction. Then, the signal amplification DNA (H2) labelled with Cy5 is used to trigger the first CHA reaction, producing the H1–H2 complex while releasing the target miRNA-21. The displaced miRNA-21 can initiates a subsequent cycle of the CHA cascade. After each CHA reaction is complete, the hybridization of H2 and H1 causes the AuNPs to move away from the UCNPs. Therefore, FRET is inhibited, leading to UCL recovery of UCNP. The stable fluorescence of Cy5 at 670 nm and the changing UCL of the UCNPs at 540 nm constitute a ratiometric fluorescent biosensor, effectively eliminating environmental interference and greatly improving the sensitivity and detection stability of the fluorescence sensor.

## 2 Experimental

### 2.1 Materials

YCl_3_·6H_2_O, YbCl_3_·6H_2_O, ErCl_3_·6H_2_O, TmCl_3_·6H_2_O, oleic acid (OA), 1-octadecene (ODE), methanol, sodium hydroxide, cyclohexane, ammonium fluoride (NH_4_F), HAuCl_4_, phosphate-buffered saline (PBS), 2-(*N*-morpholino)ethanesulfonic acid (MES), tris(2-carboxyethyl)phosphine hydrochloride (TCEP–HCl), 1-ethyl-3-(3-dimethylaminopropyl)carbodiimide hydrochloride (EDC), trisodium citrate (C_6_H_5_Na_3_O_7_), and *N*-hydroxysuccinimide (NHS) were obtained from Aladdin (Shanghai, China). All the oligonucleotide sequences utilized in this study were obtained from Sangon Biotechnology Co., Ltd (Shanghai, China). The sequences of the DNA and RNA employed in this study are listed below:

miRNA-21: 5′-UAGCUUAUCAGACUGAUGUUGA-3′

miRNA-214: 5′-UGCCUGUCUACACUUGCUGCUGC-3′

miRNA-141: 5′-UAACACUGUCUGGUAAAGAUGG-3′

miRNA-155: 5′-UUAAUGCUAAUCGUGAUAGGGGU-3′

miRNA-1: 5′-UGGAAUGUAAAGAAGUAUGUAU-3′

miRNA-499: 5′-UUAAGACUUGCAGUGAUGUUU-3′

H1: 5′-(SH) TTTTCAACATCAGTCTGATAAGCTACATGTGTAGTAGCTTATCAGACT(NH_2_)-3′

H2: 5′-(Cy5) ATAAGCTACTACACATGTAGCTTATCAGACTGATGCATGTGTAG-3′

### 2.2 Equipment

The morphology and structure were examined *via* transmission electron microscopy (TEM) on a JEM-2100F equipment with an accelerating voltage of 200 kV. Powder X-ray diffraction (XRD) patterns were recorded on a Miniflex600 diffractometer (Rigaku, Japan). UV-vis absorption spectra were measured with a Lambda 950 spectrophotometer. Fourier transform infrared (FTIR) spectra were acquired on a Nicolet 5700 spectrometer. Zeta potential measurements were conducted on a Nano-ZS90 analyzer (Malvern Instruments). Emission and excitation spectra were measured *via* a FluoroMax-4 spectrofluorometer. Upconversion luminescence spectra were acquired *via* excitation with a laser system at 980 nm.

### 2.3 Preparation and modification of the UCNPs

#### 2.3.1 Preparation of NaYF_4_:20%Yb,4%Er,0.1%Tm

NaYF_4_:20%Yb,4%Er,0.1%Tm nanoparticles were synthesized in the light of a formerly reported assay.^44^ Briefly, 230.6 mg of YCl_3_·6H_2_O, 77.5 mg of YbCl_3_·6H_2_O, 15.3 mg of ErCl_3_·6H_2_O, and 3.8 mg of TmCl_3_·6H_2_O were dissolved in 5 mL of methanol. Subsequently, 6.0 mL of OA and 15.0 mL of ODE were introduced. The reaction system was initially heated to 110 °C, subsequently elevated to 160 °C and held for 30 min. After cooling to 40 °C, a methanol solution of 148.2 mg of NH_4_F and 100 mg of NaOH was added dropwise. The temperature was elevated to 110 °C with continuous stirring to evaporate the methanol completely, followed by heating to 300 °C for 1.5 h crystallization step.

#### 2.3.2 Preparation of NaYF_4_:20%Yb,4%Er,0.1%Tm@NaYF_4_:20%Yb

Core–shell UCNPs were fabricated through a seed-mediated growth approach.^45^ Specifically, 242.6 mg of YCl_3_·6H_2_O and 77.5 mg of YbCl_3_·6H_2_O were added to the mixture containing 15.0 mL of ODE and 6.0 mL of OA. The mixture was heated to 110 °C and then cooled to 40 °C. A methanol solution of NH_4_F (148.2 mg) and NaOH (100 mg) was added dropwise. Subsequently, 10.0 mL of a cyclohexane dispersion containing NaYF_4_:20%Yb,4%Er,0.1%Tm core nanocrystals was injected. After stirring at 40 °C for 30 min to facilitate shell deposition, the reaction temperature was gradually increased to 110 °C with continuous stirring to evaporate the methanol completely, followed by annealing at 300 °C for 1.5 h to yield core–shell nanostructures. The UCNPs were washed sequentially with ethanol and water *via* centrifugation and finally stabilized in cyclohexane for storage.

#### 2.3.3 Modification of the UCNPs

The conversion of OA-UCNPs to citrate-modified UCNPs *via* the ligand exchange method. In a typical ligand exchange process, 588.2 mg of trisodium citrate and 15.0 mL of diethylene glycol were mixed. The solution was thermally processed to 110 °C, maintained for 30 min, and then controlled cooling to 60 °C. Subsequently, 10 mg of OA-UCNPs dispersed in 5.0 mL of toluene were added dropwise. The temperature was elevated to 130 °C to evaporate the cyclohexane and toluene residues, followed by reheating to 180 °C with magnetic stirring for 1.0 h to complete ligand exchange. The Cit-UCNPs were purified through triple centrifugation with ethanol and water and redispersed in ultrapure water for subsequent use.

### 2.4 Preparation of AuNPs

AuNPs were synthesized *via* the citrate reduction assay.^46^ Briefly, 100 mL of ultrapure water and 20 µL of HAuCl_4_ were mixed. The reaction mixture was vigorously stirred at 600 rpm with magnetic agitation and heated to boiling. Subsequently, 5 mL of 0.034 M trisodium citrate solution was infused into the refluxing reaction system. The reaction was kept under vigorous ebullition for 10 min until the color of the reaction mixture altered from pale yellow to wine-red. After termination of heating, the colloidal suspension was cooled to ambient temperature and preserved at 4 °C for subsequent applications.

### 2.5 Construction of a ratiometric fluorescent probe

500 µL of the Cit-UCNP aqueous dispersion was mixed with 3000 µL of MES buffer (10 mM, pH 5.5). Under continuous stirring, 3 mg of EDC and 6 mg of NHS were added to activate surface carboxyl groups for 30 min. Afterwards, 12 µL of H1 solution (10 µM) was introduced, and the reaction system was incubated at 36 °C for 2.5 h. The resulting UCNP–H1 conjugates were isolated by centrifugation (12 000 rpm, 8 min) and redispersed in 600 µL of PBS buffer (10 mM, pH 7.4) for storage at 4 °C. The conjugation efficiency of the UCNPs with H1 was calculated as:^47^1
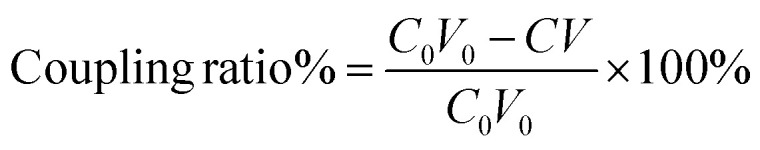
2
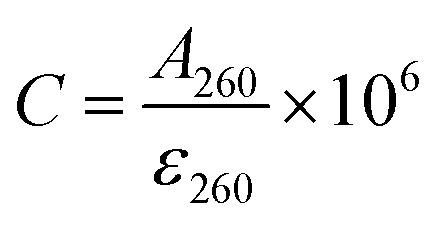
where *C*_0_ represents the concentration of H1 added to the reaction mixture, *V*_0_ represents the volume of the reaction mixture, *C* represents the concentration of H1 in the supernatant after centrifugation and collection after coupling is complete, *V* represents the volume of the supernatant, and *A*_260_ represents the supernatant. The absorbance at 260 nm, *ε*_260_ represents the molar extinction coefficient. 200 µL of the UCNP–H1 conjugates were reacted with 10 µL of TCEP–HCl (10 mM) at 36 °C for 2 h in a thermomixer to reduce disulfide bonds. Then, 125 µL of the colloidal AuNP mixture was added, and the mixture was incubated with shaking at 36 °C for 16 h. After controlled hybridization annealing, the UCNP–H1–AuNP nanoprobes were obtained.

### 2.6 Fluorescence measurement

A 350 µL aliquot of the UCNP–H1–AuNP nanoprobe mixture was mixed with 1 µL of 20 nM H2. Subsequently, 1 µL of target miRNA-21 was added to acquire final concentrations of 0.25, 0.5, 1.25, 2.5, 5, 12.5, 25, and 50 pM. The mixture was diluted to 400 µL with PBS (10 mM, pH 7.4) and incubated at 36 °C under light-protected conditions for 105 min. Fluorescence intensity was measured under 980 nm excitation using a calibrated spectrofluorometer. In accordance with the literature,^48^ a sensitivity calibration curve was established by plotting the ratio of UCNP-to-Cy5 fluorescence intensity (dependent variable) against the miRNA-21 concentration (independent variable). In reference to the literature,^49^ the limit of detection (LOD) was calculated according to the following formula: LOD = 3*σ*/*S*, where *σ* represents the standard deviation of the fluorescence intensity from ten blank measurements and S denotes the slope of the sensitivity calibration curve. Each experiment was repeated three times, and the solutions without miRNA-21 were continuously detected for 10 times.

### 2.7 Analysis of actual samples

Prior to miRNA-21 detection with the nanoprobes, the serum specimens were subjected to 20-fold dilution with PBS buffer (10 mM, pH 7.4). The target miRNA-21 in the diluted serum matrix was quantified *via* the standard addition method. The subsequent detection procedures for the serum samples followed identical protocols depicted in Section 2.6.

### 2.8 Statistical analysis

Each experiment above involved three replicates. This setup ensures the statistical robustness of the data and accounts for both inter-experimental and intra-experimental variations. Data analysis, including ANOVA, standard curve plotting, and correct interpretation, was performed. All the data are presented as the means ± standard deviations. The experimental data were analyzed and plotted. Statistical significance was evaluated *via* one-way ANOVA followed by Tukey's *post hoc* test. The regular pattern was used to establish the significance levels. *P < 0.05, **P < 0.01, ***P < 0.001.

## 3 Results and discussion

### 3.1 Characterization of the resultant UCNPs

**Fig. 1:**
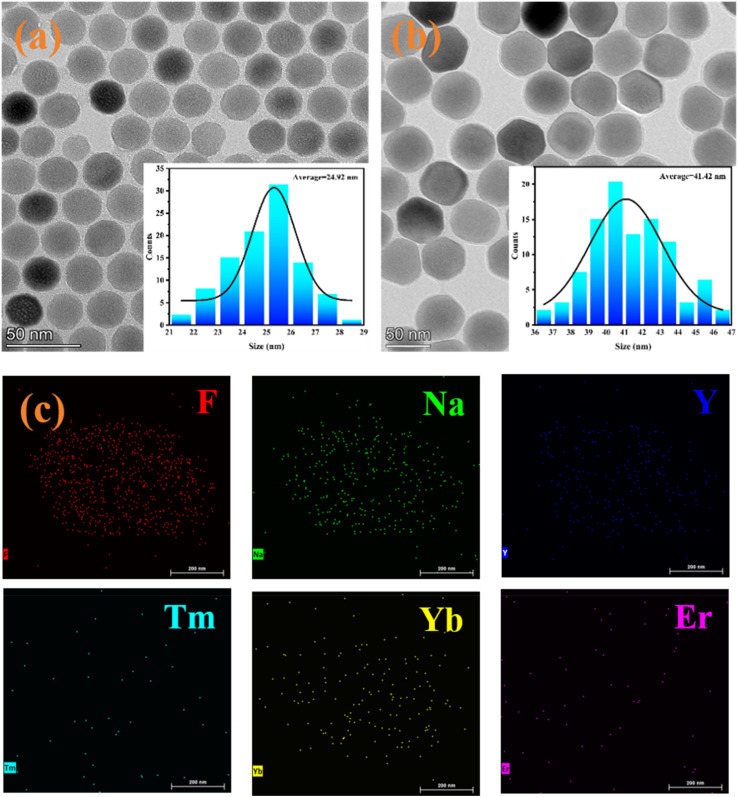
(a) TEM image of the NaYF_4_:20%Yb,4%Er,0.1%Tm nanoparticles; (b) TEM image of the NaYF_4_:20%Yb,4%Er,0.1%Tm@NaYF_4_:20%Yb nanoparticles; (c) EDS images of the NaYF_4_:20%Yb,4%Er,0.1%Tm@NaYF_4_:20%Yb nanoparticles.

In this study, a novel FRET-based sensitive probe for the quantification of miRNA-21 was constructed with UCNPs as energy donors. Therefore, NaYF_4_:20%Yb,4%Er,0.1%Tm (the core UCNP) and NaYF_4_:20%Yb,4%Er,0.1%Tm@NaYF_4_:20%Yb (the core–shell UCNP) nanoparticles were first synthesized *via* a solvothermal method. TEM, XRD, FT-IR, UV-vis, and zeta potential tests were employed to observe the shape, size, crystal form, and surface functionalization of the obtained nanomaterials. As shown in Fig. 1a, the morphologies of the synthesized NaYF_4_:20%Yb,4%Er,0.1%Tm nanoparticles are highly monodispersed hexagonal crystals with an average particle size of 24.92 nm. In contrast, the resulting NaYF_4_:20%Yb,4%Er,0.1%Tm@NaYF_4_:20%Yb nanoparticles still retained a similar structure; however, the average particle size increased to 41.42 nm due to shell formation (Fig. 1b). In addition, elemental analysis of the obtained nanoparticles was performed *via* EDS, and Fig. 1c shows that obvious elements, such as Er, Y, Yb, Tm, F and Na, can be observed. These analytical results indicate that the core–shell UCNPs were fabricated and that all the elements were uniformly dispersed.

**Fig. 2:**
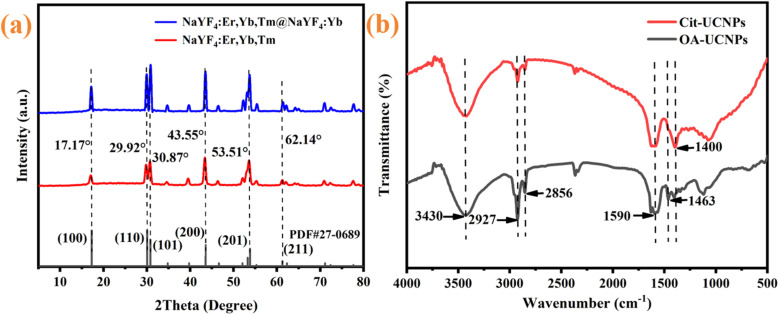
(a) XRD pattern of the nanoparticles; (b) FT-IR spectra of the OA-UCNPs and Cit-UCNPs.

To verify the crystalline purity and hexagonal structure of the synthesized nanomaterials, the XRD patterns of the as-prepared nanomaterials were obtained. As shown in Fig. 2a, all the diffraction peaks of the nanoparticles indexable to hexagonal NaYF_4_ (JCPDs No. 27-0689), and absence of the cubic phase or other impurities is observed. This result indicates that the synthesized nanoparticles are highly crystalline and hexagonal, which is consistent with the TEM results. To enhance the hydrophilicity of the UCNPs and broaden their biomedical applicability, the surface of the UCNPs was functionalized with sodium citrate to obtain Cit-UCNPs. The surface structure of the UCNPs before and after functionalization was characterized by FT-IR spectroscopy. As illustrated in Fig. 2b, the specific absorption at 3430 cm^−1^ represents the stretching vibration of –OH groups, the specific absorption at 2927 and 2856 cm^−1^ are attributed to the asymmetric and symmetric stretching vibrations of –CH_2_– groups, and the specific absorption at 1590 and 1463 cm^−1^ are the diagnostic peaks of the asymmetric stretching vibrations of the –COO^−^ group and methylene bending vibration, respectively, demonstrating the presence of OA on the surface of the UCNPs. After modification with citrate, the methylene specific absorption of OA molecules corresponding to 2927 cm^−1^ and 2856 cm^−1^ became weak, and the peak at 1463 cm^−1^ even disappeared, indicating a significant decrease in the content of lipophilic and hydrophobic long alkyl chains, and the vibrational frequency of carboxyl groups migrated to 1400 cm^−1^. FT-IR analysis demonstrated that citrate was effectively functionalized on the surface of the UCNPs through ligand exchange to obtain Cit-UCNPs.

**Fig. 3:**
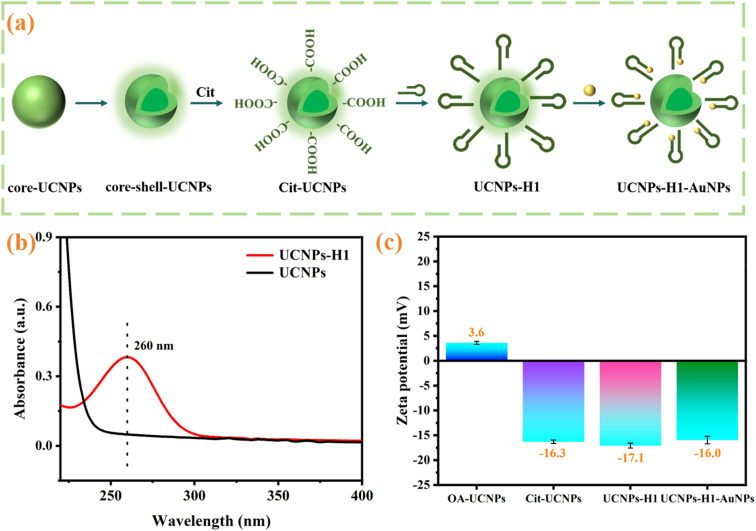
(a) Surface modification steps; (b) UV-vis spectra of the UCNPs and UCNP–H1; (c) zeta potentials of the OA-UCNPs, Cit-UCNPs, UCNP–H1 and UCNP–H1–AuNPs.

A number of surface modification steps were subsequently carried out to achieve water solubility and covalent attachment of DNA sequences on the surface of the UCNPs (Fig. 3a). The H1 modifications of the UCNPs were first verified *via* UV-vis absorption spectroscopy (Fig. 3b). After H1 functionalization, the UV-vis spectra of the UCNPs display a characteristic absorption at 260 nm, verifying that H1 has successfully functionalized the surface of the UCNPs. Furthermore, the surface charges of the nanomaterials were determined *via* a zeta potential analyzer (Fig. 3c). OA-UCNPs are positively charged, with a zeta potential of 3.6 mV. After modification with citrate, the zeta potential of the UCNPs changed to −16.3 mV, confirming that the carboxyl groups were effectively incorporated into the UCNPs. The zeta potential of UCNP–H1 shifted to −17.1 mV, which was attributed to the negative charge carried by the phosphate group in the H1 single chain. These results indicated that H1 was successfully conjugated on the surface of the UCNPs. In addition, the coupling efficiency of UCNP to H1 was calculated according to Section 2.5, and the conjugation efficiency was 52%, which further indicates that H1 has been successfully bound to the surface of UCNP and can be used to further construct fluorescent biosensing systems.

### 3.2 Characterization of AuNPs

**Fig. 4:**
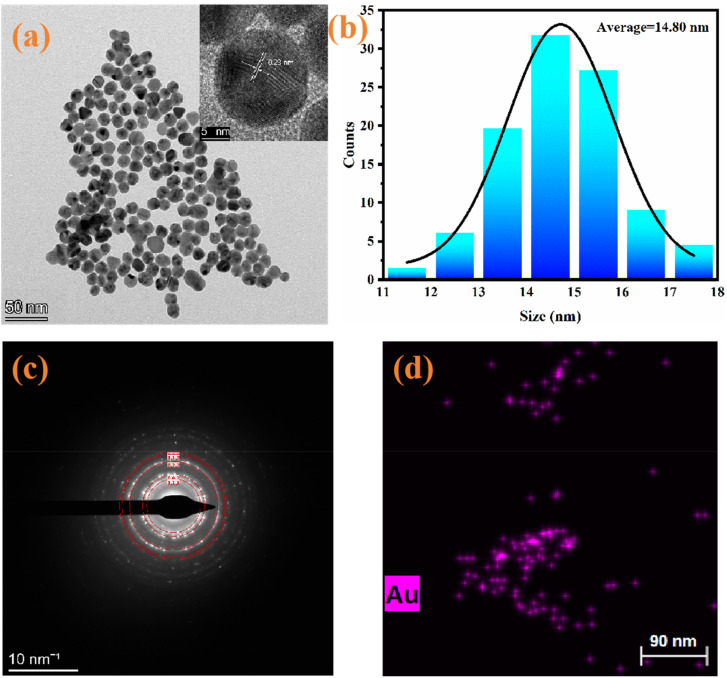
(a) TEM image and high-resolution crystal plane analysis of AuNPs; (b) particle size distribution of AuNPs; (c) electron diffraction pattern of the AuNPs; (d) element distribution of AuNPs.

AuNPs can effectively quench fluorescence through an energy transfer phenomenon. Here, we used AuNPs as energy acceptors because they have many excellent properties, such as tunable optical properties, facile surface functionalization, excellent biocompatibility, and high extinction coefficients. AuNPs were first synthesized through the citric acid reduction method and then characterized *via* TEM, electron diffraction patterns and EDS. The TEM image and particle size distribution (Fig. 4a and b) revealed that the synthesized AuNPs were uniformly spherical, and the average size was approximately 14.80 nm. Moreover, the illustration in Fig. 4a reveals the lattice fringes of the AuNPs, with a lattice spacing of 0.23 nm, which is in agreement with the (111) lattice spacing of Au. As shown in Fig. 4c, the concentric circle distribution presented in the electron diffraction pattern reflects the multicrystalline structure of the AuNPs. The ring with the highest diffraction intensity corresponds to the (111) crystal plane of the face-centered cubic (FCC) gold crystallite, whereas the other diffraction rings correspond to the (200), (220), and (311) crystal planes. The distribution pattern is consistent with the FCC standard diffraction pattern. As shown in Fig. 4d, elemental analysis was conducted on the AuNPs, revealing the presence of Au. The above data indicate that AuNPs were also successfully prepared.

### 3.3 Principle and feasibility of the proposed assay for the detection of miRNA-21

**Fig. 5:**
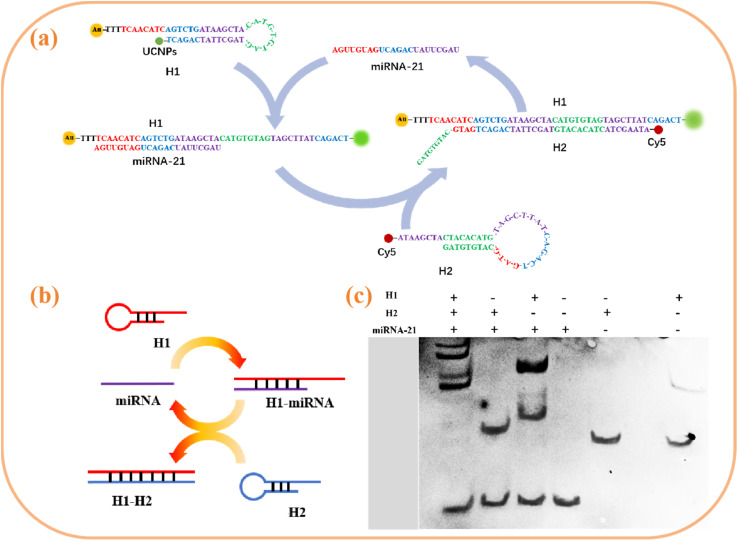
(a and b) Schematic of the CHA reaction; (c) PAGE characterization of the CHA reaction.

As illustrated in Fig. 5a and b, this study engineered a bifunctional hairpin DNA H1 (modified with thiol/amino groups at the 5′/3′ termini) that was conjugated to AuNPs and UCNPs *via* Au–S bonds and amide linkages, respectively, to construct a FRET donor–acceptor pair. The resulting assembly effectively quenched the UCL through the FRET. Upon recognition of target miRNA-21 at the loop region of H1, the strand displacement reaction exposes the concealed initiator sequence in the stem domain. This sequence hybridized with the Cy5-labelled auxiliary probe H2, triggering CHA. The initial CHA cycle generated a stable H1–H2 duplex while releasing miRNA-21, which subsequently acted as a recyclable trigger for continuous CHA amplification. Following each CHA completion, the formation of the H1–H2 duplex induced spatial separation between the AuNPs and UCNPs, abolishing the FRET effect. Consequently, the UCL of the UCNPs at 540 nm progressively recovered with increasing target concentration, whereas Cy5 emitted a stable reference fluorescence at 670 nm independent of the target concentration. This dual-channel emission strategy enabled the development of a ratiometric fluorescent biosensor that effectively eliminates environmental interference while significantly enhancing detection sensitivity and operational robustness.

Nondenaturing polyacrylamide gel electrophoresis of different oligonucleotide systems was conducted to validate the viability of the CHA process. The lanes from right to left are H1, H2, miRNA-21, H1 + miRNA-21, H1 + H2, and H1 + H2 + miRNA-21, respectively. As shown in Fig. 5c, the single-stranded DNA is longer and migrates at a slower rate, whereas the miRNA-21 fragment is shorter and migrates faster, which is reflected in the difference in lanes 1–3. Lanes 4 and 5 indicate that chain hybridization occurs between H1 and miRNA-21. When H1 and miRNA-21 were incubated, one new band with low mobility appeared, corresponding to a slower migration rate, which is shown as three bands. H2 cannot hybridize with miRNA-21 and is shown as two bands. Three bands appear in lane 6, and the first band from the top has a slower migration rate than the first band in lane 4. This finding indicates that after miRNA-21 was introduced into H2, it hybridized with H1 through the CHA reaction to form a double-stranded structure with more base pairs, reducing the migration rate. The above electrophoresis experiments indicate that the CHA strategy can be used.

**Fig. 6:**
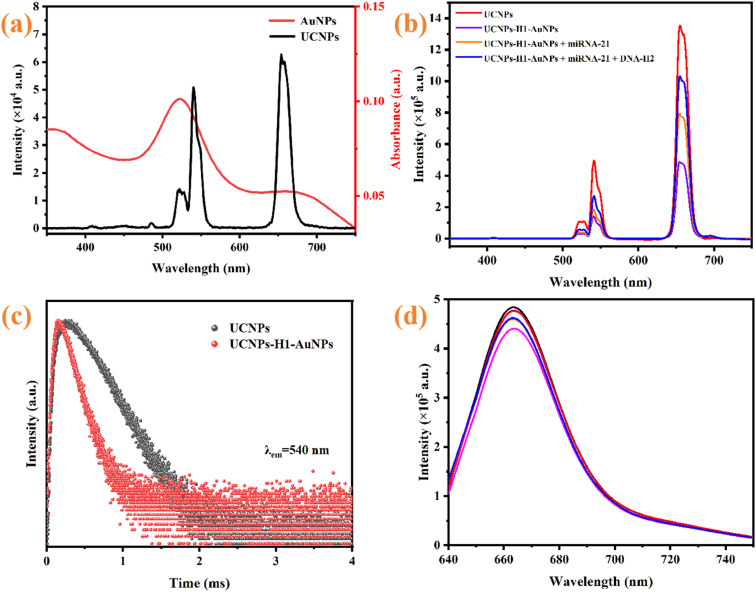
(a) Emission characteristics of the UCNPs and optical absorption of the AuNPs; (b) fluorescence responses of different test systems; (c) fluorescence lifetime decay curve; (d) fluorescence spectra of Cy5.

To investigate the UCL suppression mechanism and feasibility of the AuNPs on the UCNPs, the UV-vis absorption spectra of the AuNPs and the UCL spectra of the UCNPs were first characterized. As presented in Fig. 6a, the AuNPs have significant surface plasmon resonance absorption peaks in the 450–600 nm range, whereas the UCNPs exhibit strong UCL in the 500–600 nm band. The two spectra show a good overlap region, which provides a necessary prerequisite for the FRET process. The fluorescence spectrum analysis in Fig. 6b revealed that the UCL intensity of the detection system significantly decreased when UCNP–H1 and AuNPs were linked. However, after the analyte miRNA-21 was added, the UCL intensity was restored. Moreover, the UCL intensity was significantly greater after the addition of H2 than after the addition of only the target miRNA-21, indicating that CHA signal amplification was amplified.

Further verification experiments were conducted through fluorescence lifetime analysis, and the quenching influence of AuNPs on the fluorescence lifetime of the UCNPs was detected *via* time-resolved fluorescence spectroscopy. As shown in Fig. 6c, the fluorescent decay times of the UCNPs before and after the addition of AuNPs were 589 µs and 238 µs, respectively. This attenuation feature is consistent with the typical characteristic of a shortened donor lifetime in the FRET mechanism.^50^ The above results confirm that energy transfer can be achieved between the UCNPs and AuNPs through the FRET pathway. In addition, there was no significant change in the fluorescence intensity of Cy5 at 670 nm during the detection of the target miRNA-21 (Fig. 6d), indicating that Cy5 has a good internal reference effect on the constructed sensor. Therefore, a ratiometric fluorescent probe based on the stable fluorescence of Cy5 at 670 nm and the changing UCL of the UCNPs at 540 nm can effectively eliminate environmental interference and significantly enhance the analyticalsensitivity and detection stability of the fluorescence probe.

### 3.4 Optimization of detection conditions for miRNA-21

**Fig. 7:**
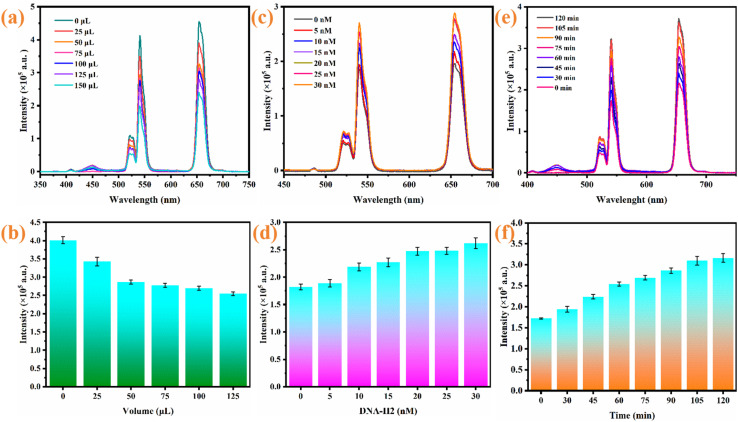
(a and b) AuNP quantity-dependent fluorescence response; (c and d) H2 concentration-dependent emission modulation; (e and f) temporal evolution of fluorescence intensity.

Prior to the detection of miRNA-21, the key operational parameters including the amount of AuNPs, the dosage of H2 and the reaction time were optimized. Fig. 7a and d show that the UCL intensity decreased and reached the lowest value when the amount of AuNPs was 125 µL. Therefore, 125 µL of AuNPs was determined as the ideal amount. Fig. 7c and d illustrate that the UCL signal exhibits progressive enhancement as the H2 dosage elevates and stabilizes when the H2 dosage surpasses 20 nM. Therefore, the concentration of H2 used in subsequent experiments was 20 nM. Finally, the UCL intensities of the detection system at different reaction times (0–120 min) were measured. As shown in Fig. 7e and f, the UCL intensity gradually increased with increasing reaction time and achieved signal stabilization at 105 min, which reflects that the CHA reaction reached completion. Thus, 105 min was established to be the ideal reaction time.

### 3.5 Analytical performance of the developed assay

**Fig. 8:**
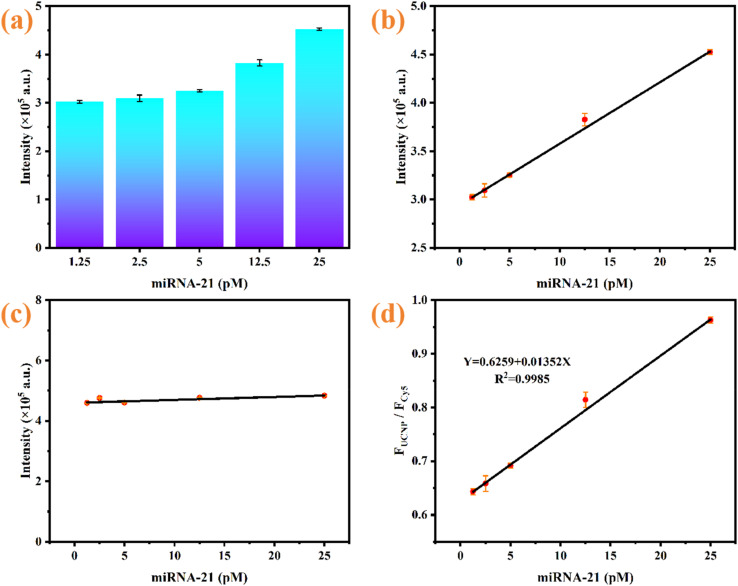
(a and b) UCL intensity changes of the sensing system at 540 nm under various concentrations of miRNA-21; (c) fluorescence intensity changes of Cy5 at 670 nm under various concentrations of miRNA-21; (d) quantitative correlation between *F*_UCNP_/*F*_Cy5_ and different concentrations of miRNA-21.

The sensitivity of the developed approach for miRNA-21 quantification was measured under optimized experimental conditions. Fig. 8a and b show that the UCL intensity at 540 nm gradually recovered with increasing concentrations of miRNA-21, indicating that the specific binding of the target miRNA-21 with the nanoprobe can effectively trigger UCL recovery. Fig. 8c shows that as the concentration of the target miRNA-21 increased, the luminous intensity of Cy5 at 670 nm changed very little, indicating that Cy5 has a good role as an internal reference. Therefore, a quantitative calibration curve was constructed, and there was a significant linear correlation between the fluorescence intensity ratio (*F*_UCNP_/*F*_Cy5_) and the concentration of miRNA-21 in the range from 0.5 to 25 pM (Fig. 8d). According to the description in Section 2.6, the established sensitivity equation is *Y* = 0.6259 + 0.01352*X* (*R*^2^ = 0.9985), where *Y* denotes the ratio of fluorescence intensity between UCNP and Cy5 and *X* denotes the miRNA-21 concentration. The LOD was calculated to be 0.28 pM on the basis of the 3*σ* rule. Furthermore, the detection capability of the proposed assay was benchmarked with that of previous studies. As illustration in Table 1, the LOD of this work is lower than that of several previously reported approaches, which may attribute to the CHA signal amplification and the high FRET efficiency between the UCNPs and AuNPs. In contrast to enzyme-dependent systems requiring complex reactions, our probe employs CHA-based enzyme-free amplification, which streamlines operational procedures and minimizes interference risks during target recognition and signal transduction. Moreover, compared with other fluorescent materials, UCNPs excited by near-infrared light serve as fluorescent donors, reducing the occurrence of false positive signals with less background radiation, while exhibiting excellent and stable optical performance. These findings confirmed that the designed sensing platform has exceptional analytical characteristics for the measurement of miRNA-21.

**Table 1:** Comparison of detection performance for miRNAs

Material	Amplification strategy	Time (min)	Medium	Linear range	LOD (pM)	Ref.
AuNP/MnO_2_	DNAzyme	120	Plasma	0.1–10 nM	44	51
ZIF-8	DNAzyme	—	—	0.1–1 nM	47.85	52
FAM/BHQ-1	EDC dual-end DNAzyme	90	Serum	0.01–1 nM	2	53
ZnO NPs	DNAzyme	120	—	0.1–30 nM	54	54
FAM/BHQ-1	HCR + DNAzyme	60	Tissue/cell	0.01–0.2 nM	3	55
UCNP/AuNP	CHA	105	Serum	1.25–25 pM	0.28	This work

**Fig. 9:**
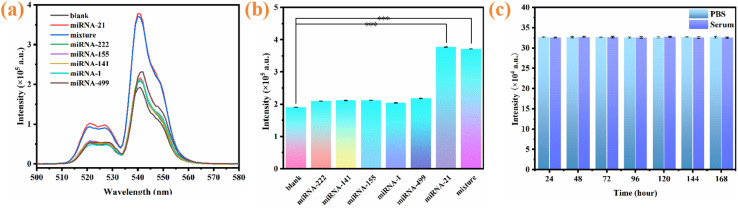
(a and b) Effects of other miRNAs on UCL; (c) fluorescence intensity stability test within different times.

The selectivity of the proposed approach was assessed by using miRNAs with similar structures and associations with acute myocardial infarction, such as miRNA-222, miRNA-141, miRNA-155, miRNA-1, miRNA-499 and miRNA-21.^56^Fig. 9a and b show that the UCL intensities corresponding to the five other miRNAs are significantly lower than those corresponding to miRNA-21, indicating that the five other miRNAs cannot cause noticeable UCL recovery in the detection system. Therefore, the developed assay has exceptional discrimination capability for miRNA-21 detection among phylogenetically-related miRNAs. This exceptional selectivity originates from the high-affinity hybridization between miRNA-21 and the H1 probe. To evaluate the operational stability of the constructed fluorescent biosensor systematically, miRNA-21 detection was performed at 24-hour intervals over a 7-day period. As shown in Fig. 9c, the sensor maintained consistent detection responses in both PBS buffer and serum matrices, demonstrating its robust performance across these critical media. Furthermore, the sensor retained its detectable capability after multiple experimental sets, confirming its excellent operational stability.

### 3.6 Analysis of miRNA-21 in serum samples

To assess actual sample applicability, the biosensor's performance was evaluated in serum matrices spiked with standardized miRNA-21 concentrations. The serum matrices were fortified with miRNA-21 at three different concentrations (1.4 pM, 6.0 pM, and 12 pM). By measuring the fluorescence responses and correlating them with the established calibration curve, we quantified the miRNA-21 concentrations and calculated the recovery rates. As displayed in Table 2, the quantitative analysis of serum samples spiked with three different concentrations of miRNA-21 was obtained with recoveries ranging from 94.3–102.7%. These results demonstrated the reliable performance of the developed assay for miRNA-21 detection in biological samples.

**Table 2:** Quantitative analysis of miRNA-21 in serum *via* the developed approach

Samples	Added (pM)	Found (pM)	Recovery (%)	RSD (%, *n*= 3)
Serum	1.4	1.32 ± 0.011	94.3	0.87
	6	5.81 ± 0.447	96.8	1.98
	12	12.32 ± 0.115	102.7	3.63

## 4 Conclusions

This study has constructed an high-performance and sensitive sensing platform for miRNA-21 determination employing target-triggered CHA signal amplification and FRET between UCNPs and AuNPs. This sensor integrates the signal amplification of the CHA reaction and the anti-Stokes shift UCL property of the UCNPs to amplify the detection signal and enhance the signal-to-noise ratio. In addition, a ratiometric fluorescent biosensor was constructed by using the fluorescence intensity of Cy5 at 670 nm as an internal reference and combining the changes in the UCL intensity of the UCNPs at 540 nm, eliminating the influence of environmental conditions by determining the ratio of fluorescence intensities at 670 nm and 540 nm. Under the optimized experimental conditions, a determination concentration interval of 1.25–25 pM and a detection limit of 0.28 pM were obtained. This study achieved highly sensitive and interference-resistant detection of miRNA-21; however, limitations remain, including the requirement for complex sample pretreatment and the lack of multitarget detection capability. Future work should focus on enhancing the clinical utility of the detection system by rationally leveraging the multiband UCL of UCNPs. This approach would enable simultaneous analysis of multiple biomarkers while exploring applications of fluorescent probes in live-cell imaging.

## Ethical statement

Human serum samples were attained from North Campus of Zhongda Hospital Affiliated to Southeast University. Before the laboratory study, written informed consent was signed by all volunteers. All animal procedures were performed in accordance with the Guidelines for Care and Use of Laboratory Animals of North Campus of Zhongda Hospital Affiliated to Southeast University and approved by the Animal Ethics Committee of North Campus of Zhongda Hospital Affiliated to Southeast University.

## Author contributions

Tingman Xu, Chengke Huang and Tiantian Ge designed research. Tingman Xu and Chengke Huang performed the experiments. Songsong Song and Yanyi Tan analyzed data. All author wrote and revised the manuscript.

## Conflicts of interest

The authors declare that they have no known competing financial interests or personal relationships that could have appeared to influence the work reported in this paper.

## Data Availability

The authors confirm that the data supporting the findings of this study are available within the article.
